# Investigating COVID‐19 stress and coping: Substance use and behavioural disengagement

**DOI:** 10.1002/ijop.12820

**Published:** 2021-11-18

**Authors:** Esther Greenglass, Daniel Chiacchia, Lisa Fiskenbaum

**Affiliations:** ^1^ Department of Psychology York University Toronto ON Canada

**Keywords:** COVID‐19 anxiety, Coping, Behavioural disengagement, Substance use

## Abstract

The purpose of this online empirical study was to examine the relationship between COVID‐19 stress, coping including substance use and behavioural disengagement, and avoidance behaviour early on in the COVID‐19 pandemic. Participants, recruited from Amazon's Mechanical Turk (MTurk, *N* = 730), were adults from Canada, the United States, Italy, Germany and the United Kingdom. Results of path analysis showed that feeling threatened by the virus, predicted greater COVID‐19 anxiety, which was related to greater substance use to cope with the virus, as well as more behavioural disengagement, which predicted less avoidance behaviour. Implications of the results are discussed, particularly the relationship between coping and avoidance behaviour during the pandemic.

COVID‐19 has rapidly emerged as a worldwide pandemic and within a short period of time has caused widespread illness, deaths, economic disruption and hardship. On 11 March 2020, the World Health Organization (WHO) declared a global pandemic of COVID‐19 (World Health Organization [WHO], 2020). The Introduction begins with a discussion of stress and the pandemic followed by occupational risk and coronavirus, theoretical factors, coping, self‐efficacy, avoidance behaviour and then, the theoretical model put forth in this study.

## Stress and the pandemic

Stress associated with COVID‐19 is unparalleled. Uncertainty and fear increased significantly during the pandemic and can be related to stress (Codagnone et al., 2021). Bogliacino et al. (2021) and Codagnone et al. (2020) investigate the mechanisms through which COVID‐19 and negative shock impact cognitive capacity, preferences, fears and expectations. There are examples from many countries highlighting a widespread deterioration in mental health. For example, Canadians were experiencing a decline in their mental health and coping due to COVID‐19 (Jenkins et al., 2021) and Ogrodniczuk et al. (2021) found that 79.3% of Canadian men indicated that COVID‐19 negatively affected their mental health. Anxiety screenings, which consist of individuals' responses to online questions about self‐reported mental illness symptoms were 93% higher in the United States than pre‐pandemic levels (Mental Health America, 2020) and over 40% of the population in Italy and the United Kingdom were at risk for mental illness, defined as the conditional probability of being under high stress, anxiety and depression given economic vulnerability and negative economic shock (Codagnone et al., 2020).

## Occupational risk and coronavirus

People in some occupations are more susceptible to the virus than others (Lu, 2020) because these occupations require person‐to‐person contact, which puts those workers at risk for exposure to the virus (Olya, 2020), thus resulting in an increase in stress. Occupations, such as service and domestic work, restaurant work, retail, tourism and hospitality, for example, require face‐to‐face interactions with others and thus present a heightened risk for contracting the virus. Because of the nature of these jobs, working from home is often not an option. To the extent that one's work requires employees to be in close physical contact with others, perceived occupational risk (associated with COVID‐19) should increase, as well as stress.

## Theoretical factors and coping

Theoretically, stress appraisal is a concept that can bridge the gap between a stressor and psychological health. According to the *Transactional Theory of Stress* (Lazarus & Folkman, 1984), stressors are indirectly related to emotional outcomes, such as anxiety, through cognitive appraisal. Specifically, stressors are *primarily appraised* by evaluating whether or not the stressful encounter has the potential to cause harm or danger (i.e., threat). If stressors are appraised as threatening, they are then *secondarily appraised* by evaluating one's resources to manage the stressor which may include self‐efficacy and coping (Hobfoll, 1988). Coping strategies are conceptualised in different ways and a common dichotomy is grouping coping strategies into problem‐focused and emotion‐focused (Lazarus & Folkman, 1984). While problem‐focused coping can alter the stressor by direct action, emotion‐focused coping strategies are focused on regulating internal emotional states, that is, making one feel better. In general, emotion‐focused coping is associated with emotional and behavioural problems while problem‐focused coping is related to more positive adjustment and fewer personal problems (Seiffge‐Krenke & Klessinger, 2000). According to theory, emotion‐focused coping is most likely to occur when an appraisal has been made that nothing can be done to modify harmful or threatening environmental conditions (Lazarus & Folkman, 1984). Since the pandemic is ongoing and cannot be controlled by the individual, the predominant coping form during COVID‐19 should be emotion‐focused. In recent research COVID‐19 results in several negative shocks defined as losses of income, assets and health (Bogliacino et al., 2021; Bogliacino & Montealegre, 2020). Integrating the experience of stress with cognitive functioning, this research shows that the effects of negative shocks, due to COVID‐19, have taxed cognitive function, as documented by experimental and correlational evidence.

In the present research, two emotion‐focused coping forms in relation to the pandemic will be examined: Behavioural disengagement and substance use. Behavioural disengagement is a maladaptive coping style that reflects the tendency to reduce one's efforts in coping with persistent and difficult situations and can result in the person giving up coping because the stress is overwhelming. With the pandemic altering every aspect of life, it would not be surprising to find many individuals using behavioural disengagement to cope with stress. Additionally, substance use, that is, the use of alcohol and drugs, as a way of coping, has increased during the pandemic (Wardell et al., 2020). Further, research has shown that substance use, as a way of coping with stress, is greater in males than in females (NIDA, 2020). Moreover, alcohol and drug‐taking behaviour are maladaptive since they are negatively related to psychological well‐being and positively related to stress (Carver, 1997). Since substance use regulates one's emotional reactions to a stressor, that is, it makes people feel better temporarily, rather than managing the stressor itself, substance use should be associated with greater behavioural disengagement from the problems associated with the pandemic. The use of alcohol and other drugs may function as a coping strategy after other attempts to cope have been tried and failed to reduce stress, a plausible explanation given to the ongoing and chronic nature of the pandemic. While men use substances more to cope with stress, anxiety symptoms are more common in women (Nolen‐Hoeksema et al., 1999).

Self‐efficacy is another resource that people may use when dealing with stress. People with high self‐efficacy believe they have the ability to manage prospective situations and exercise influence over them and self‐efficacy is associated with lower stress (Jerusalem & Schwarzer, 1992). Therefore, high self‐efficacy should be associated with less threat and lower COVID‐19 anxiety.

## Gender differences, stress and coping

Research conducted prior to COVID‐19 indicates that there are gender differences in stress and anxiety as well as in coping behaviours. For example, women have been shown to have higher levels of perceived stress than males (Aparisi et al., 2019) and anxiety symptoms are more common in women (Nolen‐Hoeksema et al., 1999). Other research demonstrates that men use substances more to cope with stress than do women (Nolen‐Hoeksema et al., 1999). Therefore, it is expected that women should report greater anxiety related to COVID‐19 than men. It is further expected that substance use, including the use of alcohol and drugs, would be greater in men than in women.

## Avoidance behaviour

During a pandemic, there are various behaviours that can reduce the risk of infection such as, wearing a mask, using hand sanitiser, frequent hand washing and avoiding large gatherings of people found at sporting events, shopping malls and large holiday celebrations. In this research, we focus on avoidance behaviour since frequent reports link attendance at large gatherings with spikes in infection (Courtemanche et al., 2020). It is worth noting that avoidance behaviour in this context differs from the more well‐defined avoidance coping. It is possible to document several ways in which people can be found gathering with others. These include travel, eating in restaurants and attending concerts or sporting events. Since COVID‐19 is transmitted among people, the public has been advised to avoid places where there were large gatherings of people. In order to control the spread of infection, governments have implemented public health measures prohibiting large gatherings of people. These measures resulted in closure of recreation programmes, libraries, private schools, daycares and places of worship, as well as bars and restaurants, except for takeout or delivery. Measures varied in frequency depending on the numbers of new infections in a given jurisdiction or city at a particular time. However, people do not always comply with these measures. According to Piltch‐Loeb et al. (2017), when analysing compliance with public health recommendations, a combination of knowledge, risk perception and efficacy should be considered. Moreover, there should be a relationship between the coping strategies people use to manage their stress and avoidance behaviour during a pandemic. This is consistent with previous findings that frequent use of adaptive coping strategies and infrequent use of maladaptive coping strategies are associated with positive health outcomes (Lo Buono et al., 2017). When the coping strategy people use is focused more on regulating one's emotional reactions to the stressor and not on the stressor itself, the person's behaviour would be less effective in managing a stressor according to the Transactional Theory (Lazarus & Folkman, 1984). To the extent that individuals use maladaptive coping strategies, that is, the use of alcohol and drugs, as well as behavioural disengagement, they should become less vigilant and therefore would be less likely to avoid large groups, that is, avoidance behaviour.

## Theoretical model

**Figure 1:**
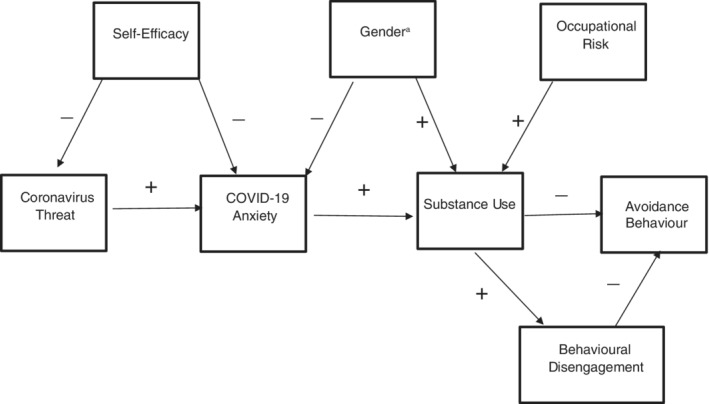
Theoretical model: COVID‐19 anxiety, substance use and avoidance behaviour. ^a^1. Female; 2. male.

In light of the above, a theoretical model is put forth (Figure 1): Coronavirus threat should be positively related to COVID‐19 anxiety, which should predict increased substance use. Substance use should predict behavioural disengagement. Self‐efficacy in dealing with coronavirus should predict less coronavirus threat and less COVID‐19 anxiety. Males should report greater substance use than females, and females should report greater COVID‐19 anxiety. Greater occupational risk of getting the virus should predict more substance use. Lastly, substance use and behavioural disengagement should predict less avoidance of high‐risk situations, that is, avoidance behaviour.

The model was tested with participants from five different countries; Canada, the United States, the United Kingdom, Italy and Germany. The countries included in the study are approximately representative of how well the pandemic has been managed as reflected in the number of COVID‐19 cases/per 1 million population (Worldometer, 2021). Of the five countries, Canada had the lowest number of cases (35,549), followed by Germany (43,418) and then the United Kingdom (65,363), then Italy (69,361), with the most number of cases/per 1 million population found in the United States (101,774).

## METHODS

### Procedure and participants

Data were collected in March and April 2020 which allowed assessment of psychological reactions to COVID‐19 early on when it was first declared a pandemic (WHO, 2020). Participants were 730 adults^1^ recruited on *Amazon's Mechanical Turk* (*MTurk*),^2^ a tool for collecting data online that has been used reliably by various disciplines including social psychology (Eriksson & Simpson, 2010). The survey was administered in English and was designed to ensure reading comprehension of the participants^.^ Each participant was paid 1 USD Most participants were 18 years old or older. Once selected, participants then proceeded to the study's questionnaire posted on *Qualtrics*, where they were assigned a randomly generated number that allowed for identification for payment purposes. The data from this project have been placed on the *Open Science Framework* (https://osf.io/rs5ke/; DOI: 10.17605/OSF.IO/RS5KE^3^).

In the combined sample, the average age of participants was 32.50 years old (*SD* = 10.34). Thirteen participants were removed from the data due to non‐conscientious responding (Marjanovic et al., 2014). Thirty‐five percent (*N* = 256) were female and 40% (*N* = 293) were married or common law. A little more than two‐thirds of the total sample (*N* = 511) were university‐educated including undergraduate and graduate education. Thirty‐five percent (*N* = 261) responded that their occupation presented risks of getting COVID‐19 (Table 1).

**TABLE 1:** Demographic statistics by country

Variable	Canada	United States	United Kingdom	Italy	Germany	Total
*N*	144	153	148	140	144	729
Age Mean (SD)	32.99 (9.55)	37.54 (11.79)	31.39 (10.25)	30.87 (9.71)	29.39 (8.04)	32.50 (10.34)
Mean (*SD*)	32.99 (9.55)					
Female, % (*N*)	42 (61)	38 (58)	40 (59)	33 (46)	21 (31)	35 (256)
Married or common law, % (*N*)	55 (80)	42 (65)	43 (64)	33 (47)	25 (37)	40 (293)
Education, % (*N*)						
High school	16 (23)	23 (35)	19 (29)	41 (58)	31 (45)	26 (190)
Trade school	2 (4)	6 (10)	4 (6)	1 (2)	4 (6)	3 (28)
University	81 (118)	70 (108)	75 (112)	57 (80)	64 (93)	70 (511)
Occupational risk						
“Not at all,” % (*N*)	34 (50)	38 (59)	34 (51)	33 (46)	38 (55)	35 (261)

### Measures

Table 2 presents a summary of the measures, authors, sample items and Cronbach's alphas, most of which were greater than .80.

**TABLE 2:** Study variables and Cronbach's alphas

Variable	Author	Sample item	Cronbach's alpha
Coronavirus threat	Chiacchia et al. (under review)	How much do you feel at risk {about coronavirus}	.84
Avoidance behaviour	Authors	Avoid large gatherings of people	.91
Behavioural disengagement	Carver (1997)	I've been giving up trying to deal with it {coronavirus}	.82
Substance use	Carver (1997)	I've been using alcohol or other drugs to help me get through it {coronavirus}	.90
Self‐efficacy	Adapted from, Jerusalem and Schwarzer (1992)	I am confident that I can deal efficiently with it {coronavirus}	.78
COVID‐19 anxiety	Shacham (1983)	Indicate your recent feelings about coronavirus… “tense”	.92
Occupational risk^a^	Authors	Does your occupation present risks for your getting coronavirus?	—

^a^ Single item.

The Brief Coronavirus Threat Scale (BCTS) (Chiacchia et al., under review), adapted from the Financial Threat Scale (Marjanovic et al., 2013), was used to assess coronavirus threat due to coronavirus. Participants were asked to indicate how they felt about each of five statements by selecting a response alternative that ranged from 1, not at all, to 5, extremely/a great deal (Table 3).

**TABLE 3:** The Brief Coronavirus Threat Scale (BCTS) (Chiacchia et al., under review)

Indicate how you feel about the coronavirus by answering the following questions^a^						
		Not at all				Extremely/A great deal
1.	How uncertain do you feel?	1	2	3	4	5
2.	How much do you feel at risk?	1	2	3	4	5
3.	How much do you feel threatened?	1	2	3	4	5
4.	How much do you worry about it?	1	2	3	4	5
5.	How much do you think about it?	1	2	3	4	5

^a^ The Coronavirus Threat Scale score is computed by calculating the mean response to five items.

*Avoidance behaviour scale*, composed by the authors, consists of 10 items assessing avoidance of situations considered high risk for infection such as large gatherings of people. Participants were asked to indicate the extent to which they had avoided each situation by selecting a response that went from 1, not at all to 5, a great deal.

*Behavioural disengagement* (Carver, 1997) consisted of two items that measure the extent to which individuals had given up dealing with the virus. Response alternatives went from 1, I haven't been doing this at all, to 4, I've doing this a lot.

*Substance use* (Carver, 1997) consisted of two items measuring extent to which alcohol and other drugs were used to deal with coronavirus. Response alternatives went from 1, I haven't been doing this at all, to 4, I've doing this a lot.

*Self‐efficacy* is a four‐item measure that is adapted from Jerusalem and Schwarzer (1992). Participants indicated how much they could manage coronavirus by selecting the alternative that reflected how true each statement was for them, with response alternatives from 1, not at all true, to 4, exactly true.

COVID‐19 *anxiety* is a six‐item measure (POMS: Shacham, 1983) assessing the degree of anxiety associated with coronavirus thus referring to COVID‐19 anxiety; responses went from 1, not at all, to 5, extremely.

*Occupational risk* is a one‐item measure composed by the authors that assesses degree of risk of getting coronavirus the participant's occupation presents with response alternatives from 1, not at all, to 4, very much so. See Table 4 for means, range and standard deviations for study variables.

**TABLE 4:** Composite study variables: Total sample

Variable	N	Mean	SD	Range
Coronavirus threat	730	3.30	.85	1–5
Self‐efficacy	730	2.92	.53	1–4
COVID‐19 anxiety	730	2.69	1.03	1–5
Avoidance behaviour	730	4.22	.90	1–5
Substance use	730	1.46	.78	1–4
Behavioural disengagement	728	1.52	.76	1–4

### Analytic plan

Analysis of variance was conducted on mean coronavirus threat scores across countries. This is followed by a correlation matrix of all study variables. Path analysis was used to examine the relationships among study variables in the theoretical model with the total sample using AMOS Version 15 (Arbuckle & Wothke, 1999) since the variables were observed (not latent) and were not hypothetical constructs (Ullman & Bentler, 2003). The model was not tested in each country for statistical power. Although there is little consensus on the recommended sample size for path analysis, Garver and Mentzer (1999) proposed a “critical sample size” of 200. That is, as a rule of thumb, any number above 200 provides sufficient statistical power for data analysis. Since none of the national samples in this study had a sample size approaching 200, we combined the data to get a total *N* of 730.

### Ethical compliance

All procedures performed in this research were in accordance with the ethical standards of the Human Participants Review Sub‐Committee, York University's Ethics Review Board (certificate number: 2020‐102) and with the 1964 Helsinki Declaration and its later amendments or comparable ethical standards. Informed consent was obtained from all individual adult participants included in the study.

## RESULTS

Results of the analysis of variance conducted on mean coronavirus threat scores indicated that there were statistically significant differences across countries (*F* (4, 725) = 7.72, *p* < .001, η ^2^ = .041). Tukey's Honest Significant Difference post‐hoc tests revealed that mean scores were significantly lower in Germany than in Canada (*p* < .001), the United States (*p* < .01), the United Kingdom (*p* < .05) and Italy (*p* < .001). An evaluation of these effect sizes indicated that the standardised mean difference was largest when comparing Germany and Canada; coronavirus threat in Germany was significantly lower than in Canada. Although the confidence interval was fairly wide, it captured a moderately large effect, thereby suggesting that the true population mean difference may be substantial (see Table 5 for effect sizes by country).

**TABLE 5:** Standardised mean differences (i.e., Cohen's *d*) of coronavirus threat with 95% confidence intervals by country

	Canada	United States	United Kingdom	Italy	Germany
Canada	**3.51 (0.76)**	0.22 [−0.12, 0.45]	0.29 [0.06, 0.52]	0.17 [−0.06, 0.40]	0.65 [0.41, 0.88]
United States		**3.33 (0.88)**	0.07 [−0.16, 0.29]	0.06 [−0.29, 0.17]	0.39 [0.16, 0.62]
United Kingdom			**3.27 (0.88)**	−0.13 [−0.36, 0.10]	0.33 [0.09, 0.56]
Italy				**3.38 (0.77)**	0.48 [0.24, 0.48]
Germany					**2.99 (0.85)**

*Note*: Country‐level means and standard deviations (in parentheses) on the diagonal in bold.

Table 6 reports a correlation matrix of all variables. Results show that coronavirus threat correlated positively with COVID‐19 anxiety, substance use, avoidance behaviour and behavioural disengagement. Self‐efficacy, in dealing with coronavirus, correlated negatively with COVID‐19 anxiety and coronavirus threat. COVID‐19 anxiety was positively related to substance use, avoidance behaviour, behavioural disengagement, occupational risk and being female. Substance use was positively related to behavioural disengagement, occupational risk and being male. Substance use and behavioural disengagement were negatively related to avoidance behaviour. Behavioural disengagement was positively related to occupational risk (Table 6).

**TABLE 6:** Correlation matrix of study variables (*N* = 730)

Variable	CT	COVID‐19 anxiety	SE	SU	AV BH	BD	OR^a^	Gender^b^
Coronavirus threat (CT)	**—**	.63***	−.32^***^	.20^***^	.18^***^	.17^***^	.14^***^	−.18^***^
COVID‐19 anxiety		**—**	−.38^***^	.28^***^	.10^**^	.33^***^	.15^***^	−.18^***^
Self‐efficacy			**—**	−.02	−.06	−.06	.06	.12^**^
Substance use				**—**	−.14^***^	.42^***^	.10^**^	.10^**^
Avoidance behaviour					**—**	−.16^***^	−.01	−.07^*^
Behavioural disengagement						**—**	.12^**^	−.01
Occupational risk							**—**	.00
Gender								**—**

AVBH = avoidance behaviour; BD = behavioural disengagement; CT = coronavirus threat; SE = self‐efficacy; SU = substance use.

^a^ OR = occupational risk 1, not at all, 4, very much.

^b^ Gender 1. Female 2. Male.

* *p* < .05.

** *p* < .01.

*** *p* < .001.

### Results of path analysis

Several fit indices were used to evaluate the fit of the model to the data. A model is considered to have an acceptable fit with the data if the χ^2^ statistic (chi‐square test) is non‐significant. However, given the sensitivity of the chi‐square statistic to sample size, a number of alternative fit measures are generally used as well. Specifically, The Tucker Lewis Index (TLI), the Comparative Fit Index (CFI), the Goodness of Fit Index (GFI) and the Adjusted Goodness of Fit Index (AGFI) should be greater than .95 for an adequate fit and the root mean square error of approximation (RMSEA) should be less than .08 (Hu & Bentler, 1999).

**Figure 2:**
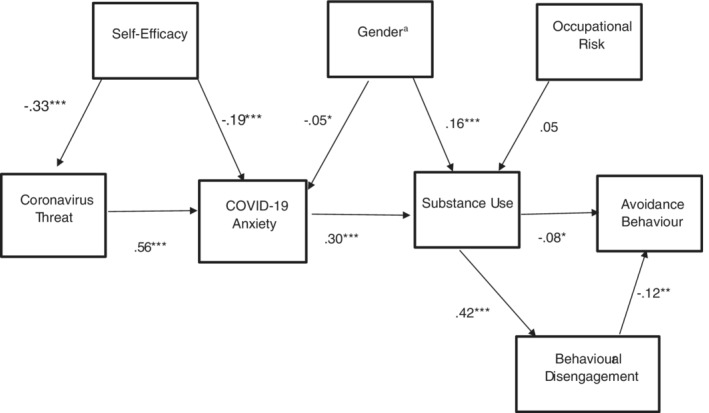
Empirical model I: COVID‐19 anxiety, substance use and avoidance behaviour: Standardised path coefficients. *Note*: Although the standardised regression coefficients for paths from gender to COVID‐19 anxiety, and occupational risk to substance use, were the same (i.e., .05), their standard errors were different (.06 for gender to COVID anxiety and .026 for occupational risk to substance use). Consequently, the regression coefficient from gender to COVID‐19 anxiety is significant, and the regression coefficient from occupational risk to substance use is not. ^a^1. Female; 2. male. *p < .05. **p < .01. ***p < .001.

In the empirical model presented in Figure 2, the chi‐square was significant (χ^2^(18) = 148.684, *p* < .001) which could be due to the large sample size (*N* = 726). The GFI = .95, AGFI = .90, CFI = .84, TLI = .77 and the RMSEA was .10, thus indicating that the hypothesised model was not a satisfactory fit with the data. Examination of the standardised path coefficients shows that self‐efficacy was negatively related to threat and COVID‐19 anxiety. Being male and COVID‐19 anxiety were associated with more substance use. Being female was related to greater COVID‐19 anxiety. Avoidance behaviour decreased with substance use and behavioural disengagement. No significant relationship was found between occupational risk and substance use. Two modification indices were indicated: coronavirus threat to avoidance behaviour, and COVID‐19 anxiety to behavioural disengagement. Modification indices were considered in a judicious manner to attain a better fit. These paths resulted in the greatest change to the overall chi‐square model fit as seen in the next analysis.

**Figure 3:**
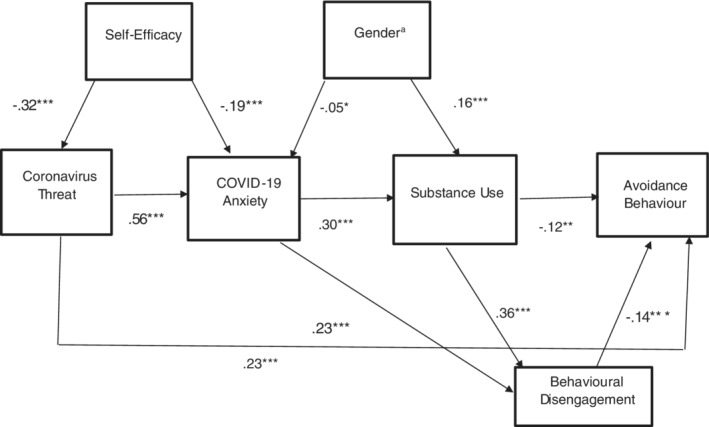
Empirical model II: COVID‐19 anxiety, substance use and avoidance behaviour: Standardised path coefficients. ^a^1. Female; 2. male. *p < .05. **p < .01. ***p < .001.

The model was rerun with the two modification indices and the nonsignificant path from occupational risk to substance use was dropped. The fit indices indicated that our hypothesised model was a good fit with the data (χ^2^(10) = 43.707, *p* < .001, GFI = .98, AGFI = .95, TLI = .92, CFI = .96 and RMSEA = .06). Examination of the standardised regression coefficients shows that self‐efficacy was associated with lower threat and lower COVID‐19 anxiety which led to greater disengagement and more substance use. Being male was associated with more substance use, being female was associated with more anxiety and greater substance use was associated with less avoidance behaviour and more disengagement. Behavioural disengagement led to less avoidance behaviour. Coronavirus threat led to more avoidance behaviour (Figure 3).

## DISCUSSION

Findings in this study show that COVID‐19 is associated with anxiety and maladaptive coping such as substance use and behavioural disengagement. Examination of national differences in coronavirus threat indicated that it was significantly lower in Germany than in any of the other four countries. German participants may have perceived lower threat of the virus since Germany's fatality rate was lower than that in most other countries at the time of data collection, and thus may provide an explanation for German participants' perceptions of lower threat due to COVID‐19. For example, at the beginning of April 2020, Germany had 1584 deaths due to COVID‐19, which was 1.6% of their infections, compared to 12% of infections in Italy, 10% in Spain, France and Britain, 4% in China and close to 5% in Canada (Jackson, 2020). One explanation for Germany's low fatality rate is that they had been testing more people than most other countries. Thus, they could identify more people with few or no symptoms, thereby increasing the number of known cases, but not the number of fatalities. Early testing also allowed the German authorities to slow the spread of the pandemic by isolating known cases while they identified infections. Therefore, German participants may have been less likely to perceive coronavirus as a threat to their own health than participants in other countries where deaths due to COVID‐19 were higher. These findings highlight the importance of interpreting results within the national and social context at the time of testing for the virus. National differences in the other variables were not examined here since these variables, in contrast to coronavirus threat, were conceptualised as individual person variables within each country.

While an advantage of this study is that data collection was at the international level, one of the limitations of the design was that, although data collection occurred at the outset of the pandemic, the five countries examined in the study were still in different stages of the COVID‐19 pandemic during the time of data collection. This may have introduced another source of variation when the data were combined for data analysis. The rationale for combining the countries is that data collection took place at roughly the same time for all samples, March and April 2020, which was close to the date (11 March 2020) when COVID‐19 was declared a pandemic. Another reason for combining the countries is that demographically the samples are very similar.

As predicted, findings showed gender was related to substance use as men reported more substance use, which is similar to previous findings (NIDA, 2020). Further, in this study, women reported more COVID‐19 anxiety, which is in line with pre‐pandemic gender differences that women tend to display more anxiety than men (Nolen‐Hoeksema et al., 1999). Thus, current gender differences may be an exacerbation of pre‐existing (pre‐pandemic) gender differences. Results showed a positive relationship between COVID‐19 anxiety, substance use and behavioural disengagement. Taken together, these findings suggest that when people use behavioural disengagement in the face of coronavirus threat, they are in effect giving up. This can be linked to results of previous research in which behavioural disengagement is seen as a maladaptive coping style that reflects the tendency to reduce one's efforts in coping, resulting in the person giving up (Burker et al., 2005). Our findings relating to substance use parallel findings of previous research. For example, Wardell et al. (2020) report that, when faced with high anxiety levels resulting from the pandemic, people are more likely to turn to alcohol as a way of relieving their distress. Moreover, these findings support theoretical conceptions of Lazarus and Folkman (1984) who state that when a stressor is uncontrollable by individuals, they are more likely to resort to emotion‐focused coping.

Further, findings showed that, to the extent that individuals manage their anxiety through substance use or behavioural disengagement, they were less likely to avoid situations that increased their risk of infection. Therefore, the type of coping that people use to manage stress related to COVID‐19, may put them at greater risk of contracting the disease by not engaging in avoidance behaviour. At the same time, our data show that self‐efficacy, the belief that one can successfully manage COVID‐19, is associated with less COVID‐19 anxiety. Therefore, to the extent that self‐efficacy can be increased, coronavirus stress should also decrease. This can be done through the dissemination of positive ways to cope with the virus that individuals can use to build self‐efficacy and protect themselves from getting the virus.

Findings in the present research extend theoretical conceptions of stress and coping to coping with pandemic stress. According to theory, emotion‐focused coping occurs when an appraisal has been made that nothing can be done to modify environmental conditions (Lazarus & Folkman, 1984). In this study, we focused on two emotion‐focused coping strategies, substance use and behavioural disengagement. To the extent that individuals reported greater COVID‐19 anxiety, they were more likely to report substance use and behavioural disengagement. Moreover, these maladaptive coping strategies were associated with less avoidance behaviour of large gatherings. That is, to the extent that individuals used more maladaptive coping, they were more likely to engage in risky behaviour that put them at greater risk of contracting the virus. Thus, our findings extend stress and coping theory to an understanding of behaviour during a pandemic in that maladaptive coping was associated with riskier behaviour.

One unexpected finding was that greater coronavirus threat led to avoidance of high‐risk situations, suggesting that threat may sometimes function as an alert to potential dangers to people's health. Future research on the pandemic's effects could be directed towards determining the precursors of the positive function of threats in the health sphere.

Since this study was conducted early on in the pandemic, it was not possible to use existing scales to assess coronavirus stress, see Fear of COVID‐19 Scale (Ahorsu et al., 2020) and COVID‐19 Stress Scales (Taylor et al., 2020), since they had not yet been released. Moreover, some of the measures used in the present study were not validated, including coronavirus threat, occupational risk and the avoidance behaviour measure. However, it is worth noting that coronavirus threat correlated significantly and positively with COVID‐19 anxiety and the avoidance behaviour measure had high face validity. Further, individuals were asked to voluntarily avoid large gatherings as a way of limiting the spread of the virus early on in the pandemic when data were collected for this study (in March and April 2020). However, later on, this kind of behaviour, avoiding crowds and large gatherings, became increasingly regulated by government‐enforced mandates and this varied greatly among countries when data were collected. Further, the conceptualisation of avoidance behaviour in the present study differs from avoidance coping found in the two COPE scales (Carver, 1997) used here. For example, substance use and behavioural disengagement have been considered “avoidance” in factorial studies of the Brief COPE Scales (Baumstarck et al., 2017). At the same time, only selected coping strategies were examined here, substance use and behavioural disengagement, thus limiting the ability to interpret findings in a broader context of coping strategies. In addition, factors that may have affected self‐efficacy, such as the individual's risk perception or the country's policies regarding COVID‐19, were not considered here. The cross‐sectional design of this study precluded attribution of causality to variables, and we studied *reported* behaviour rather than behaviour. Lastly, since participants were relatively young and well‐educated, we cannot generalise our findings to the general population.

### Summary and conclusions

Despite these limitations, overall, the model we developed was a good fit to the data. The paths that were tested and found significant were theoretically derived and many have been corroborated empirically in other studies. What is new here is that our findings are specific to the experience of stress during a pandemic, thus extending research and theory on stress and coping to an understanding of people's reactions to COVID‐19. At the same time, we have demonstrated that the coping strategies people may use to cope with pandemic stress can put them at increased risk of infection. There are individual differences that result in lower COVID‐19 anxiety. For example, our findings showed that higher self‐efficacy is related to lower COVID‐19 anxiety, results that are similar to previous findings that higher self‐efficacy is associated with lower stress (Greenglass & Mara, 2012). Lastly, while correlational analysis showed a positive relation between occupational risk and substance use, this relationship was non‐significant in the path analysis when other variables were considered. In conclusion, this research demonstrates the relationship between how people cope with stress due to COVID‐19, and their reported behaviour in reducing their risk of getting the virus.

## Endnotes

1 Originally *N* = 787. A total of 57 participants were deleted due to the following reasons: 19 were duplicates, one participant did not grant consent, five were from a country that differed from the five countries that were sampled, 13 were considered random responders (Marjanovic et al., 2014) and 19 did not complete the questionnaire. This left an *N* = 730.

2 Any language problems that may arise on MTurk are not unique to this platform but can also be found in cross‐cultural research as well as in surveys that include non‐native speakers (Sechrest et al., 1972). Gupta et al. (2014) advise that, when collecting data with non‐college samples, it is advisable to use simple words and sentence structures to ensure that participants understand the items. In the present study, participants' reading comprehensiveness was likely very high given that 70% of the sample was university‐educated and the questionnaire items and sentence structure were fairly simple and easy to understand.

3 For exploratory purposes we measured several other variables related to the pandemic (e.g., plans affected by the pandemic, self‐monitoring symptoms), as well as other personality, coping and affect‐related variables. However, since these variables were not relevant to our theoretical model, they were not included in the analyses.
